# Environmental Impact on Reproductive Health: Can Biomarkers Offer Any Help?

**Published:** 2017

**Authors:** Arpit Bhargava, Neelam Pathak, Radhey Shyam Sharma, Nirmal Kumar Lohiya, Pradyumna Kumar Mishra

**Affiliations:** 1- School of Biological Sciences, Dr. Harisingh Gour Central University, Sagar, India; 2- Centre for Advanced Studies in Zoology, University of Rajasthan, Jaipur, India; 3- Division of Reproductive Biology, Maternal and Child Health, Indian Council of Medical Research, New Delhi, India; 4- Department of Molecular Biology, National Institute for Research in Environmental Health, Indian Council of Medical Research, Bhopal, India

## Introduction

Environmental health is broadly defined as the aspects of human health determined by physical, chemical, biological and social factors in the environment and encompasses the assessment and control of those factors. The exogenous and endogenous environmental health determinants include all that surround us, and environmental impact includes not only physical, chemical and biological factors, but also hormones, diet and lifestyle. As most humans develop in a predictable fashion, growing from a fertilized egg to fetus, newborn, toddler, child, adolescent, and adult, there is little doubt that environment is a powerful modifier of the human reproduction and development (1). Starting 20 years ago, there has been a steady growth in the number of laboratories involved in the investigation on environmental impact on molecular reprogramming during the most critical windows of development, such as pre-conception, pre-implantation, the fetal period, and early childhood. And considerable evidence demonstrates that the gene-environmental regulation of gene expression is crucial, besides change in the DNA sequence (1, 2). The epigenetic information is often stored as chemical modifications falling into two main categories: 1- DNA methylation and 2- changes to the histone proteins that package the genome. By regulating DNA accessibility and chromatin structure, these chemical alterations influence how the genome is translated across diverse developmental stages (3). Untangling all the components of gene-environmental regulation might be problematic in conventional human setting. In this broadest sense, if contributions of multiple factors are substantial and likely to increase susceptibility to reproductive ailments, the effects of these should be apparent in the “normal” tissue prior to the development of disease. As the examination of the role of gene-environmental regulation in the developmental re-programming in humans’ remains relatively new, it is unlikely that a single approach will stand out as being more valuable or appropriate to address a potential mechanistic link (2, 3). Therefore, to yield provocative insights, cutting-edge platform technologies must be employed for studying the gene-environmental interactions in complex reproductive tract ailments that include early or delayed puberty, menstrual irregularities, infertility, sub-fertility, early pregnancy loss, fetal death, impaired fetal growth, low birth weight, premature birth, structural or functional birth defects, polycystic ovarian syndrome, endometriosis, uterine fibroids, reproductive tract infections, sexually transmitted diseases, and reproductive tract cancers. Given that developmental basis is apparently linked to adult disease outcome, there is a greater need to change our focus from treating diseases after they are detected to prevention and from risk assessment to early disease diagnosis.

### Environmental stress-induced deformities-evidence from human studies:

A differentiating tissue that is actively proliferating to generate additional cells will respond differently to an insult as compared to a tissue than a fully differentiated tissue that has already generated many of the required cells after molecular reprogramming. Evidence from both epidemiological and mechanistic studies has not only established a potential link of environmental pollutants with impaired human fertility, but also shown that they possess the ability to alter placental function and hamper fetal development. While some of these pollutants act as an endocrine disruptor, others activate a cascade of events which significantly disturbs the intact reproductive system (4). A wide range of female reproductive tract disorders like polycystic ovarian syndrome (5), placental abnormalities and aneuploidy (6) are reported to be closely associated with the environmental chemicals results. Available reports also indicate that exposure of pregnant women to air pollutants results in increased risk of different abnormalities specifically related to heart, brain and lungs. Air pollution affects the normal developmental mechanisms of fetal brain and is related to the different diseases of the central nervous system (7). The expression of two vital genes, brain-derived neurotropic factor (BDNF) and syndecan-1 (SYN1), required for normal fetal brain development decreases significantly with the increasing particulate matter 2.5 (PM2.5) exposure in utero (8). In addition, pre-natal toxic exposure interferes with fetal lung development and is associated with the higher risk of developing pulmonary disorders including asthma (9). Breton et al. demonstrated that prenatal exposure to PM10 and PM2.5 is associated with the increased carotid arterial stiffness (10). Interestingly, exposed mothers are at higher risk of giving birth to low weight babies (11). In addition to the developmental artefacts, environmental stress signals can insinuate activation of ion channels, trigger pro-inflammatory cytokine response, induce oxidative stress, and affect cellular proliferation and differentiation orchestrating myriad abnormal changes which are transferred to the daughter progenitor cells. Links between preconceptual, peri-conceptual, and prenatal toxic exposure with adult onset of non-communicable diseases such as obesity, auto-immunity, type 2 diabetes, cardiovascular disorders, cancers, neuro-degenerative and mental sickness suggests that epigenetic mechanisms such as DNA methylation, histone modifications, and micro-RNA expression might be intricately involved, however, a thorough validation in human tissue samples remains sparse.

### Biomarkers: ray of hope?:

Much of the excitement surrounding biomarkers today relates to identification of an ongoing change (genetic/epigenetic) and early disease diagnosis. For example, measuring appropriate biomarker in paternal blood and semen samples may provide early clues for well-timed and proper intervention. As significant amount of maternal chemical burden is shared with the developing fetus and infants, sampling of maternal blood or urine samples at different times of pregnancy allows an evaluation of the exposure of the fetus at different times of development, elucidating the chemicals that cross the placenta. A biomarker in clinical reproductive medicine may be useful to better understand and predict ovarian reserve, gamete quality, embryo viability and euploidy, as well as endometrial receptivity and pregnancy outcome, including miscarriage, ectopic pregnancy and obstetric complications, e.g. preeclampsia or preterm labor. Also, for effective management of *in vitro* fertilization (IVF) protocols, specific biomarkers could be used to discriminate between suitable and unsuitable embryos to be transferred. Some of the widely assessed biomarkers like serum progesterone, hCG and estrogen not only help to identify viable pregnancies but also provide crucial functional information about the disorders that affect testes, ovaries or adrenal glands. Similarly inhibin A is suggested to be beneficial in identification of ectopic pregnancies (12). Other proteins like CRP, placenta growth factor, fms-like tyrosine kinase-1(s-1FLT), leptin, transforming growth factor-a1 and plasminogen activator inhibitor may act as predictive biomarkers for different disorders including pre-eclampsia (13). Yet, these biomarkers have limited clinical exploitation due to poor sensitivity and specificity of the available methods for their detection.

### Innovative strategies:

Several thousands of environmental contaminants penetrate daily into our environment and exert various kinds of stress response on reproductive health (2). Included in the mixture of exposures are the billions of naturally occurring and man-made chemicals (*e.g*., heavy metals, persistent organic pollutants, *etc*.); physical agents such as noise, vibration, temperature, *etc*.; macro level factors like population density, sanitation, *etc*.; and a spectrum of lifestyle factors that includes diet, physical activity and sleep. Assessment of cumulative risks for any given biological end-point will involve analysis, characterization, and possible quantification of the combined risks from multiple environmental stressors. When a pollutant enters our body, it either accumulates, or is processed metabolically and excreted. For real-time monitoring, it could be the concentration of the parental compound, its metabolite or conjugated derivative with endogenous molecules via enzymatically catalyzed transformations which can be studied using mass spectroscopy techniques. On the other hand, for understanding the molecular repercussion of the exposure the mainstay of research will have to be on a combined “omics-based approach” involving bio-molecules such as DNA, RNA, proteins, and metabolites. Large scale quantification of genes coding for proteins, regulatory elements and non-coding sequences (genomics), RNA and gene expression (transcriptomics), protein expression (proteomics), metabolites and metabolic pathways (metabolomics) will enable the identification of important genes, pathways, and protein expression signatures in suitable biological matrix of sizeable cohorts of men and women (2). Previous *in vitro* and *in vivo* studies have shown that environmental exposure results in the altered metabolic profile, is vital for xenobiotic clearance thereby exposing individuals and upcoming offspring to an increased risk of different disorders including type 1 and type 2 diabetes (14). Significant changes in the chromosomal region 11p15 are reported to be associated with Beckwith Wiedemann syndrome, fetal over growth, or Silver-Russell syndrome (SRS) under growth (15). It also disturbs epigenetic mechanism which includes DNA methylation, histone modifications, and mirco RNAs (miRNAs). Earlier studies have also reported that the altered methylation of guanine nucleotide-binding protein G(s) subunit alpha isoforms short (GNASAS) and insulin (INS) is associated with the increased risk of developing coronary heart disease (16). Exposure to tobacco smoke was reported to be significantly associated with the increasing miR-223 levels in both maternal and cord blood which further co-related with the rising cotinine concentrations in maternal urine (17). Similarly, a significant down-regulation of miR-146a was observed after nicotine and benzopyrene exposure further signifying the utility of epigenetic profile in assessing exposure associated dysregulations (18). Converging lines of evidence support the possibility that epigenetics may be a causal link between the genotype and environment, and hence, between the phenotype and disease which further substantiates that the changes incurred during in utero development can be transmitted trans-generationally. Recent advances in high-throughput methylated DNA analysis through whole-genome bisulfite sequencing and RNA-Seq, epigenome-wide association studies, epigenome editing approaches are more likely to provide novel insights to developmental origins of adult diseases (2). Although the cost of these technologies might be substantially high for large-scale application at the moment, these techniques can be used for more niche questions on limited samples to gain biological insight for further exploration.

### Risk prediction, early detection and prevention:

Because of diversities in chemical nature, dose- response relationship and factors associated with susceptibility and vulnerability, design of risk assessment strategy is challenging (19). It is very likely that low and relatively innocuous concentration of contaminants often produce deleterious and reproductive health effect, which are hard to be predicted, because measurable effects are expressed only after prolonged exposure. When these incipient effects are expressed, it might be too late to take corrective actions or steps to reduce risk. Therefore, it is essential to develop biomarkers that convincingly reflect adverse exposure-response relationship. Novel biomarkers might offer vital clues for environmental-associated reproductive system diseases and thus facilitate (i) early diagnosis of a disease; (ii) recurrence or progression of a disease; (iii) identification of individuals for disease prevention; (iv) as a potential target for drugs; (v) as a marker for a drug response. Delineating the molecular signatures of environmental-associated reproductive illness requires comprehensive knowledge of the entire cascade of events from the release of an environmental contaminant through absorption, actions and damage within the body and the development of disease (20). Defining the extent and impact of exposure is a central element for understanding a complex disease form. While ascertaining the possible implication of any exogenous moiety, the precise amount that is available (internal dose) after it is absorbed via ingestion, inhalation, *in utero*, and dermal routes is only physiologically relevant. Once inside the body, the entity is transformed and either stored or eliminated, traversing through various metabolic pathways (2). Therefore, before validating a biomarker associated with any kind of environmental exposure (acute/chronic/occupational), it is necessary to discern where in this chemico-biological process the measured index comes from - that is whether it is a biomarker of exposure, susceptibility or effect? A potential biomarker can facilitate better identification of exposure, measure both susceptibility and vulnerability, and increase prediction of outcome. The scientific impact of development and validation of biomarkers in environmental-associated reproductive system diseases is highly significant as it opens the road to the exploitation, using modern-omics technologies, of thousands of biological matrices currently housed in existing bio-banks of several medical institutes. Bodily fluids have emerged as an important source of information in several acute and chronic environmentally associated pathologies (20). A number of investigators have attempted to use circulating bio-entities found in plasma, saliva, urine, milk, seminal plasma, tears, and amniotic fluid as disease biomarkers for reproductive tract ailments. These circulating entities capable of predicting the disease course include high molecular weight complexes, membrane fragments, extracellular vesicles, lipid rafts, exosomes, microvesicles, DNA, non-coding RNA and proteins. Detection of these novel entities in plasma or serum can act as a “liquid biopsy”, which can be useful for a number of biomarker applications.

### Clinical translation:

While identifying and characterizing the significant consequences of these associated factors on human reproduction and development, two defined clusters of population might be the most affected: (i) susceptible and; (2) vulnerable populations. Whereas, “susceptibility” refers to inherent biological factors, “vulnerability” indicates population at higher risk due to personal factors. Population with both susceptibility and vulnerability may together be referred as “sensitive” population. How environmental exposures affect reproductive function and development, and how this knowledge can be translated to reduce associated morbidities and enhance quality of life is precisely warranted. Recently, a large number of environmental and host risk factors have been identified that are associated with reproductive health risk resulting in dramatic fold increase in number of biomarkers reaching for clinical validation. However, the translation of these for use in the population-level screening in such a way as to have a significant impact on clinical practice poses a major challenge. Often the use of a predictive biomarker has poorer test characteristics when it is validated in two separate populations. If validated in a low disease prevalence setting, the predictive value may be low; on the other hand, in high disease prevalence setting, the same biomarker could have enough potential clinical utility. Therefore, the process of identification and validation for clinical application among the large number of purported biomarkers associated with environmental illness is a critical step in the translation process. Key to this process is to understand the differences between evaluating biomarkers and risk factors for prevention versus disease risk prediction and early detection. A robust indulgence in analysing these differences will be necessary to facilitate the translational process. The challenges are enormous but a cutting-edge tailored approach may help to set priorities for future reproductive health research, monitoring, and surveillance activities and for potential risk assessment or risk management follow-up efforts. Such strategies might pave the way to further understand the etiologic processes underlying the exposure-outcome relationship and offer possibilities for risk prediction, early detection and prevention.
